# Ontogenetic Change in the Temporal Region of the Early Permian Parareptile *Delorhynchus cifellii* and the Implications for Closure of the Temporal Fenestra in Amniotes

**DOI:** 10.1371/journal.pone.0166819

**Published:** 2016-12-01

**Authors:** Yara Haridy, Mark J. Macdougall, Diane Scott, Robert R. Reisz

**Affiliations:** Department of Biology, University of Toronto Mississauga, Mississauga, Ontario, Canada; Royal Belgian Institute of Natural Sciences, BELGIUM

## Abstract

A juvenile specimen of *Delorhynchus cifellii*, collected from the Early Permian fissure-fill deposits of Richards Spur, Oklahoma, permits the first detailed study of cranial ontogeny in this parareptile. The specimen, consisting of a partially articulated skull and mandible, exhibits several features that identify it as juvenile. The dermal tuberosities that ornament the dorsal side and lateral edges of the largest skull of *D*. *cifellii* specimens, are less prominent in the intermediate sized holotype, and are absent in the new specimen. This indicates that the new specimen represents an earlier ontogenetic stage than all previously described members of this species. In addition, the incomplete interdigitation of the sutures, most notably along the fronto-nasal contact, plus the proportionally larger sizes of the orbit and temporal fenestrae further support an early ontogenetic stage for this specimen. Comparisons between this juvenile and previously described specimens reveal that the size and shape of the temporal fenestra in *Delorhynchus* appear to vary through ontogeny, due to changes in the shape and size of the bordering cranial elements. The jugal of the juvenile specimen is tri-radiate and similar in outline with those found in other amniotes with temporal fenestrae. The available growth series of *D*. *cifellii* shows that the jugal gradually becomes a more robust, tetra-radiate element, as the proportionate size of the temporal fenestra is reduced. Ontogenetic changes of other elements that form the border of the fenestra also contribute to its reduction. This growth series provides valuable new information regarding the ontogenetic trajectory of the temporal fenestra in a Palaeozoic reptile, which may be applicable to the evolutionary event of loss of temporal fenestration in other amniotes.

## Introduction

The large diversity and exceptional preservation of terrestrial tetrapod taxa found at the Richards Spur locality, Oklahoma, USA, allows for detailed investigations of the early evolution and diversity of numerous tetrapod clades. One of the most taxonomically diverse clades found at the locality is Parareptilia, with eight recognized species [1,2].The past decade has seen the discovery and description of several new parareptilian taxa, including *Feeserpeton oklahomensis* [1], *Microleter mckinzieorum* [3], *Delorhynchus cifellii* [4], *Abyssomedon williamsi*[2], and *Colobomycter vaughni* [5]. While the fossil record of most of these taxa is restricted to one or two specimens, *Delorhynchus cifellii* is unusual in being known from several specimens, including a partial articulated skeleton [4].The Richard Spur locality has not only provided many new taxa but in the case of *Delorhynchus* also offers a wealth of information about ontogenetic variation, because of the number of distinct individuals found at various stages of ontogeny.

The genotype *Delorhynchus priscus* was first described by Fox [6], it was based on fragmentary maxillary material at a time when most of the specimens from this locality were known only from bone fragments and individual bones. Due to a new section of the Dolese Brothers Limestone Quarry being opened in 2005, the Richard Spur locality has yielded extensive new fossil material over the last decade. Numerous articulated or partially articulated skeletons have been discovered, including a second species of *Delorhynchus*, *D*. *cifellii* [4]. This new material has greatly increased our knowledge of parareptile diversity at this locality and their phylogenetic relationships within the clade, and more broadly among amniotes [4].

The new specimen of *Delorhynchus cifellii*, described here, is of particular interest because of its early ontogenetic stage compared to previously studied material [4], which grants us unprecedented insights into how various skull elements of this genus changed during growth. In order to examine cranial ontogeny in *Delorhynchus*, we compared this new specimen to all other articulated cranial material of the genus. The apparent ontogenetic changes in the temporal region also allow us to determine how the adjoining elements of the skull interact to eventually achieve the form seen in the adult. This new data promises to create a more complete view of developmental ontogeny not only in *Delorhynchus cifellii*, but also potentially in other parareptiles, and has broader implications for understanding the ontogeny of temporal fenestration in early amniotes. Overall, These comparisons permit the first detailed study of ontogenetic change in the skull of a Palaeozoic amniotes that is based on information derived from seven specimens (S1 fig).

**Institutional abbreviations**—**OMNH**, Sam Noble Oklahoma Museum of Natural History, Norman, Oklahoma, USA.

## Material and Methods

**Fig 1:**
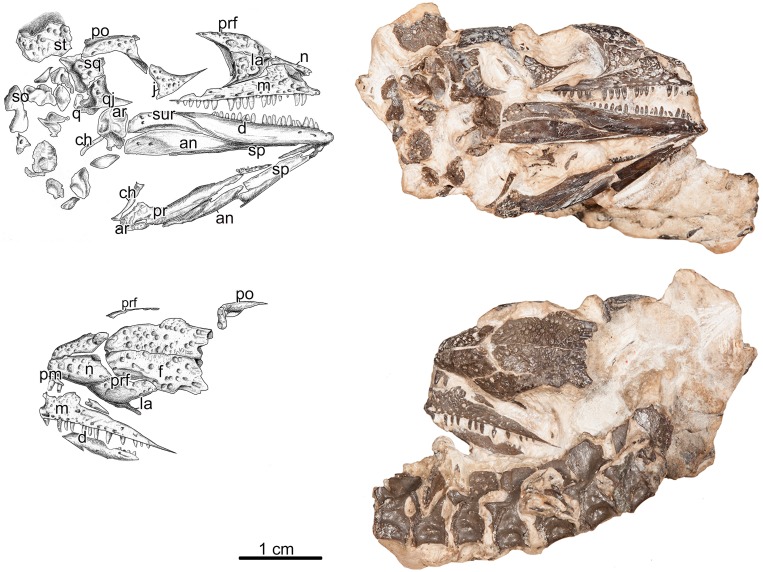
*Delorhynchus cifellii*, OMNH 77675. (A) Interpretive drawing and (B) photograph of skull and mandible in lateral view. (C) Interpretive drawing and (D) photograph of skull in dorsal view. The set of vertebra exposed on the dorsal side does not belong to this specimen. **Abbreviations: an**, angular; **ar**, articular; **ch,** ceratohyal; **d**, dentary; **f**, frontal; **j**, jugal; **la**, lacrimal; **m**, maxilla; **n**, nasal; **pal**, palatine; **pf**, postfrontal; **pm,** premaxilla; **po**, postorbital; **prf**, prefrontal; **q**, quadrate; **qj**, quadratojugal; **so,** supraorbital; **sp,** splenial; **sq**, squamosal; **st**, supratemporal. Scale bar equals 1cm.

**Fig 2:**
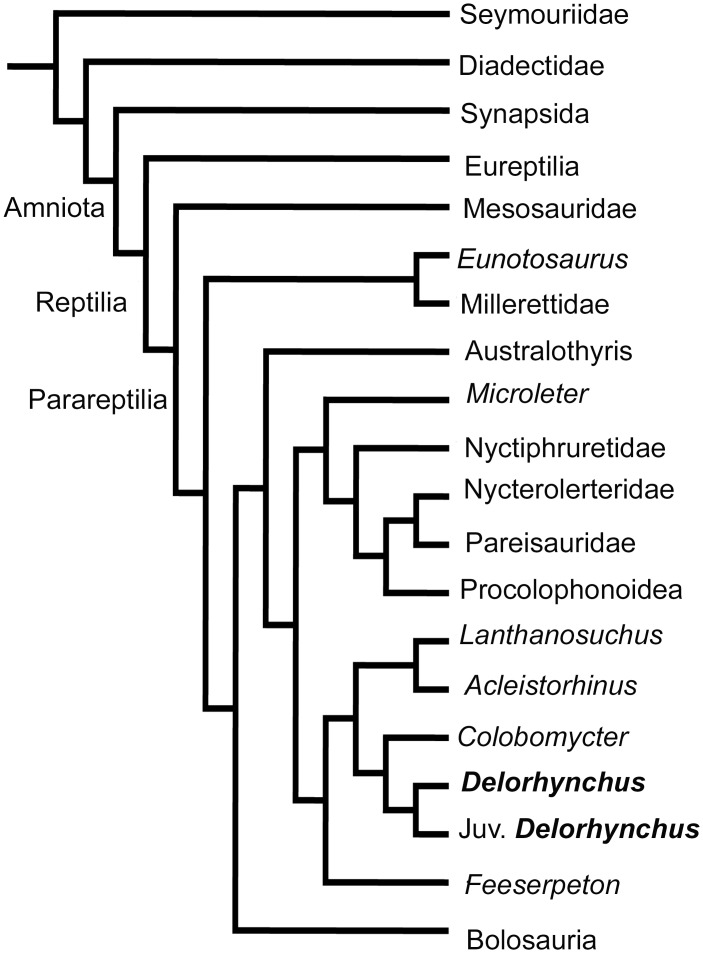
Strict consensus tree obtained from the 6 optimal trees produced by the phylogenetic analysis. The analysis places the juvenile specimen with *Delorhynchus*, supporting its attribution to the genus. Character codings of the juvenile *Delorhynchus* specimen, OMNH 77675. The specimen was added to the MacDougall et al. [7] matrix.?? 100????? ?? 101?? 000 1??????? 11? 0111 1-?11 00100 10?0??? 011 0??1?????? ????? ????? ????? ????? ????? ????? ????? ???? 0???? 1? 00????? 0–1????????? ????? ????? ????? ????? ????? ????? ????? ? 1??0

**Fig 3:**
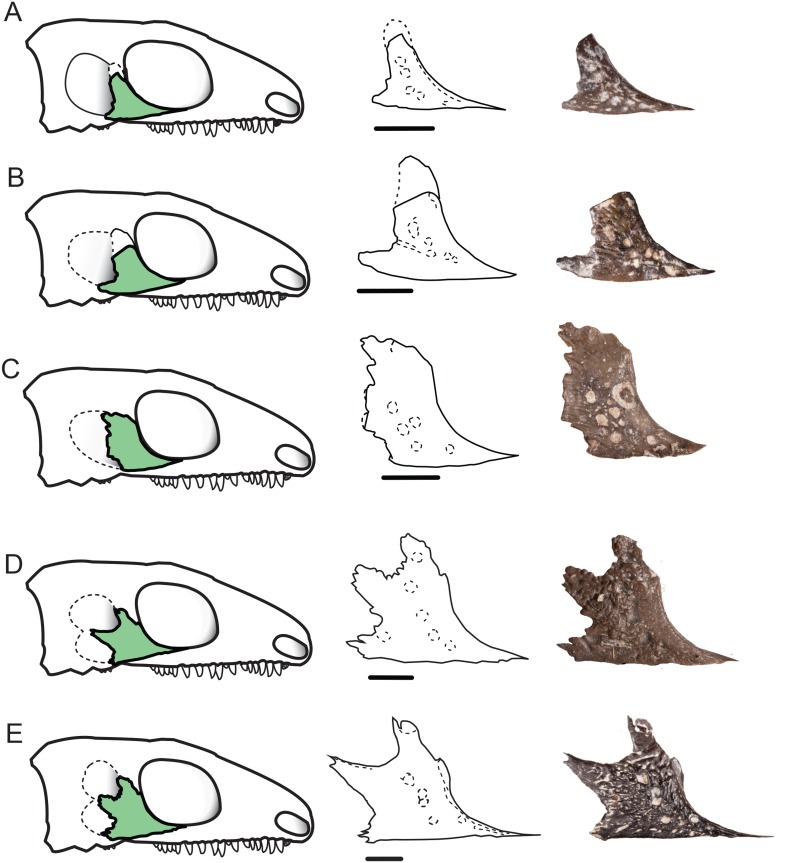
A proposed ontogenetic series featuring *Delorhynchus cifellii* exhibiting the progressive decrease in size and division of the lateral temporal fenestrae as a result of the change in jugal morphology. This series includes the following specimens: (A) OMNH 77675, (B) OMNH 77676, (C) OMNH 77677, (D) OMNH 73362, and (E) OMNH 73515. Scale bar equals 3 mm.

**Fig 4:**
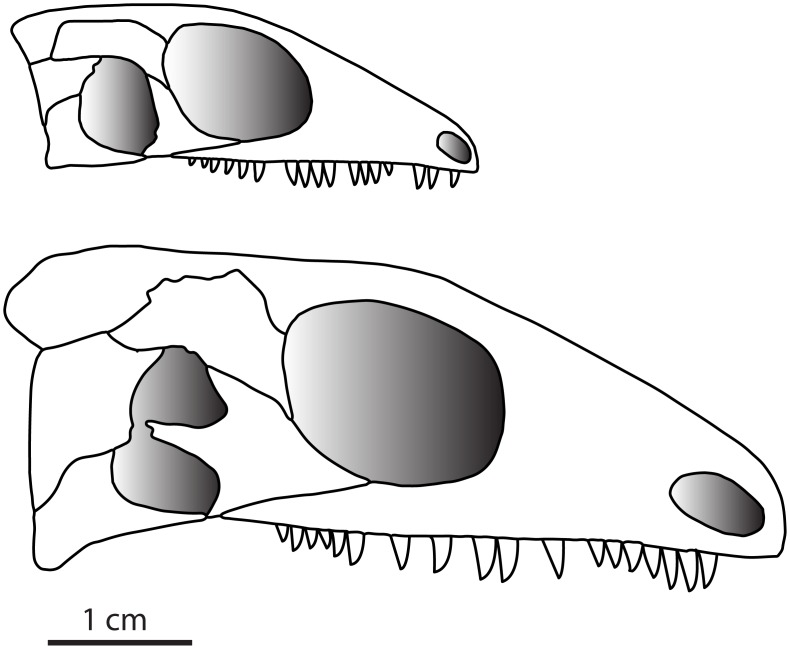
A comparison between the composite reconstructions of the youngest and most mature individuals in the growth series. Using OMNH 776575, 776576, 77677,77678, 74722, 73362, and 73515.

The specimen was prepared using pin-vices and airscribes. The illustrations were made using Adobe Illustrator CS6 to create outlines, further detail was added to Fig 1 using charcoal pencils and coquille paper. The phylogenetic analysis for Fig 2 was performed in PAUP 4.0a147, and used the data matrix of MacDougall et al. [7]. Parsimony was used as the optimality criterion, and all non-reptile taxa were designated as part of the outgroup. The comparisons made in in Fig 3 were made with five specimens assigned to *Delorhynchus cifellii*. OMNH 77675, OMNH 77676, OMNH 74722, OMNH 73362, and OMNH 73515, the latter two specimens were both previously described by Reisz et al. [4] The composite reconstructions made in Fig 4 were made using the previously listed five specimens, in addition to OMNH 77677 and OMNH 77678. All specimens used in this study are part of the collections of the Sam Noble Oklahoma Museum of Natural History, University of Oklahoma, Norman, Oklahoma, USA. No permits were required for the described study, which complied with all relevant regulations.

### Description

The newly discovered specimen OMNH 77675, described in this study, is composed of a partially disarticulated skull and mandible; elements of the posterior portion of the skull roof are missing or damaged, along with portions of the premaxilla (Fig 1). The remaining elements preserved with the specimen cannot be positively identified due to their fragmentary nature. Most of the unidentified portions of the specimen are assumed to be a combination of posterior portions of the skull roof and postcranial material. The delicate nature of this specimen, including its small size, lack of advanced dermal ornamentation that form tuberosities on the skull roof, the relatively large temporal fenestra and orbit, plus incomplete sutural fusion (Fig 1), all indicate that this is the youngest known individual of this taxon. Comparisons with other specimens of *Delorhynchus cifellii*, representing progressively more advanced growth stages, indicate that all of the morphological features that distinguish OMNH 77675 can be attributed to the immaturity of this individual.

#### Skull Roof

The small skull is widest posteriorly and tapers anteriorly into a narrow snout. This specimen exhibits a lateral temporal fenestra that is large relative to the overall size of the skull (Fig 1). The squamosal and quadratojugal border the lateral temporal fenestra posteriorly, though they are slightly disarticulated. The postorbital and the jugal are major contributors to the anterior border of the fenestra. The elements that surround the lateral temporal fenestra in *Delorhynchus* are the same as those in other lanthanosuchoids, including *Feeserpeton oklahomensis* [1], and *Acleistorhinus pteroticus* [8].

All elements of the skull roof, have distinctive ornamentation composed of deep circular pits and small shallow dimples. This basic form of ornamentation is found on the skulls of several other parareptiles, including *Microleter mckinzieorum* [3], *Feeserpeton oklahomensis* [1], *Acleistorhinus pteroticus* [8] and *Colobomycter vaughni* [5]. Previously described material of *Delorhynchus* [4] shows that there are obvious dermal tuberosities that ornament the skull roof along with the aforementioned circular pits, these tuberosities are most prevalent along the lateral edges of the skull roof elements. These tuberosities are present in all previously described specimens of *Delorhynchus* but are ontogenetically variably (S1 Fig). They are entirely absent in this new specimen (Fig 1).

The premaxilla is very fragmentary, making detailed description of this element difficult. The maxilla is a well preserved, anteroposteriorly long element that possesses an extensive number of foramina that line the ventral edge above the tooth row, with the anterior-most one being significantly larger, as is the case in most parareptiles [9]. There are 21 visible tooth positions, 18 of which are occupied by teeth. The maxillary teeth begin at the level of the external naris and extend posteriorly to the posteroventral edge of the orbit (Fig 1). All of the teeth are conical, slightly curved posteriorly, and are similar in relative size and shape to those of OMNH 73362 [4]. The dentition of this juvenile individual consists of peg shaped teeth that are slightly recurved, whereas the rest of the teeth are homodont. The large anterodorsal process of the maxilla makes contact with the nasal anteriorly and the lacrimal posteriorly. This process has a straight, unbroken dorsal edge, which is unlike the previously described larger specimens of *Delorhynchus* that show a division in this process [4]. No septomaxilla is visible in this specimen.

The nasal is a roughly rectangular element that progressively narrows anteriorly and has a distinct curvature along its lateral edges, meeting the maxilla ventrally. The nasal contributes to the dorsal margin of the external naris, whereas the maxilla borders the ventral region.

The frontal is a long rectangular element with clear doming towards the center of the bone. The dorsal surface of the frontal is exposed and well preserved in OMNH 77675 (Fig 1). The frontal contacts the nasal anteriorly and the prefrontal anterolaterally. Although the postfrontal is disarticulated from the frontal, it would have contacted the frontal along the narrow posterolateral indentation on the latter element, as seen in OMNH 73515 and OMNH 73362 [4]. In previously described *Delorhynchus* specimens (OMNH 73362, 73515) the frontal contributes to the dorsal margin of the orbit with an ornamented lateral lappet. This orbital contribution of the frontal can be seen on both sides of OMNH 77675, between the contact surface areas for the prefrontal and postfrontal bones. This orbital contribution is relatively larger in OMNH 77675 than in other specimens, but its lappet appears less well developed, indicated by the absence of tuberosities on the lappet. This is clearly a sign of immaturity, as is the shape of the interfrontal suture, which is strongly interdigitated in the more adult specimen but is nearly straight in OMNH 77675.

The lacrimal is exposed in both dorsal and lateral views, and exhibits the same ornamentation observed on the other elements of the skull roof. The bone appears to exhibit a single large, laterally exposed surface that is different from the condition in previously described specimens, which have two distinct lateral exposures [4]. This is likely because the dorsal process of the maxilla is less well developed in OMNH77675 than that found in OMNH 73362 [4], resulting in reduced overlap between the maxilla and the lacrimal, and a larger, fully exposed surface of the lacrimal.

Both prefrontal elements are preserved in this specimen, the anteroventral edge of the prefrontal contacts the lacrimal along its most dorsal surface. The anterodorsal edge of the prefrontal contacts the nasal, whereas the posterodorsal edge contacts the lateral edge of the frontal. The postfrontal in this specimen is represented by a small fragmentary piece that has been disarticulated to an extent where no useful information may be gathered.

The jugal is exposed in lateral view and has been slightly disarticulated and pushed into the orbit. It is a tri-radiate element with distinct suborbital, dorsal, and posteroventral rami (Fig 1). The suborbital ramus of the jugal is long and contacts the lacrimal anteriorly, and appears to be longer and more slender than in the other, larger specimens of *Delorhynchus cifellii*. Most of the ventral edge of the jugal meets with the dorsal edge of the maxilla along a straight suture, occupying the same position as in *Acleistorhinus pteroticus* [8]The ventral posterior process is similar to that found on the jugal of *Acleistorhinus* which also extends posteriorly to contact the quadratojugal [4,8]. The curved anterodorsal border of the jugal forms the posteroventral edge of the orbit. It is important to note that the jugal of OMNH 77675 has no posterodorsal extension like that in the *Delorhynchus cifellii* specimen described by Reisz et al. (2014). Thus, the lower temporal fenestra is large and unobstructed, this feature being attributable to the young age of the specimen. The tri-radiate jugal shape that is seen in this specimen is typical for amniotes that have lateral temporal fenestrae [10].

The postorbital in this specimen is only partially preserved and can be seen in lateral view. The lateral edges of the postorbital are nearly complete, the anteroventral process extends down to meet the jugal and simultaneously contributes to the orbital margin and the anterodorsal margin of the lateral temporal fenestra. The postorbital contacts the squamosal posteriorly and forms the dorsal edge of the temporal fenestra between the jugal and the squamosal (Fig 1). In this specimen, there is a fragment of ornamented bone that lays directly posterior to the postorbital and is identified as a portion of the supratemporal bone because it is posterior to the postorbital, as well as due to its shape.

The quadratojugal is exposed in both lateral and posterior views; the lateral surface exhibits slight sculpturing, whereas the concave posterior surface is smooth (Fig 1). Laterally, it has a long anterior and a short dorsal process, giving it the broad L-shaped outline common to lanthanosuchoids [1,4,5].The broad dorsal process meets the squamosal, whereas the slender anterior process extends forward, where it would have articulated with the jugal were it not for the slight displacement of the latter. As in all parareptiles that exhibit a lower temporal fenestra [2], the quadratojugal of this specimen contributes to both posterior and posteroventral borders of the opening (Fig 1). The occipital flange of the quadratojugal is well developed in OMNH 77675 and there is a small notch medially that contributes to the edge of the quadrate foramen.

The right squamosal is found in contact with the dorsal portion of the quadratojugal, and as with the quadratojugal, it is visible in lateral and posterior views (Fig 1). The ventral portion of the squamosal forms the posterodorsal margin of the temporal fenestra. Posteriorly, the squamosal has a smooth shelf that is continuous with the slightly concave occipital shelf of the quadratojugal.

A small portion of the quadrate is visible ventral to the squamosal and quadratojugal; little information can be garnered from it, as it is largely obscured by supportive matrix and overlying elements.

#### Mandible

This specimen preserves both mandibular rami; the left mandibular ramus is only visible in medioventral view, whereas the right mandibular ramus is exposed in lateral view. The mandible lacks the ornamentation that is found on the skull roof elements, however, there are several labial foramina found on the anterior end of the element, similar to those observed in *Colobomycter* [5,11].

The largest element of the mandible is the dentary, which spans two-thirds of the length of the mandible, and eventually tapers off above the surangular. The sutures between it and the other elements of the mandible are loosely formed, resulting in the slight separation between the dentary, surangular, and angular. The extensive interdigitation found in the larger *Delorhynchus* material described by Reisz et al. [4] is absent here. Anteriorly, the dentary is slender, and forms the entire symphysis as in many other parareptiles.

The angular is characteristically curved dorsally and overlays the surangular. The angular extends posteriorly to meet with the articular and extends farther anteriorly than is seen in the OMNH 73362, eventually underlying the splenial at about two-thirds the length of the mandible. The surangular is a curved element that contacts the articular posteriorly and tucks under the dentary and angular anteriorly.

The articular is a robust element that was not preserved in articulation on the right mandibular ramus, and is largely obscured by supportive matrix on the left ramus, although its contact with the prearticular is visible. The facet for articulation with the quadrate is visible on the right articular.

The prearticular is only visible on the left mandibular ramus, although part of it is obscured by supportive matrix dorsally. It is a long, smooth element that makes up much of the posteromedial surface of the ramus. Posteriorly, it broadens dorsoventrally to form the area that contacts with the articular (Fig 1).

The partially disarticulated state of OMNH 77675 allows for the simultaneous examination of both the exterior and interior elements of the mandible. The left ramus of the mandible displays the splenial in a clearer view than the right, which is only visible as a sliver. The splenial underlies the dentary and angular and is barely visible on the labial side. However, on the lingual side it is clear that the splenial is a smooth element that contributes extensively, along with the prearticular, to the interior surface of the mandible. Anteriorly, the splenial does not extend to the mandibular symphysis, and terminates in a small notch and a short anteroventral process, forming the Meckelian canal [12]**.**

#### Hyoid Apparatus

There are two long and slender elements near the palatal region that are likely the ceratohyals (Fig 1). This identification is based on their position in relation to the skull, their size and overall shape, and their similarity to the ceratohyal elements that are found in the holotype of *Delorhynchus cifellii* [4,5].

#### Palate

There is evidence of palatal teeth being fully formed and present; however, the delicate nature of this specimen made preparation of the underlying palatal elements impossible. The exposed denticulate ridge of the quadrate ramus of the pterygoid is similar to that seen in lanthanosuchid parareptiles, but no other palatal features can be inferred from this specimen.

#### Phylogenetic analysis

In order to test whether this juvenile specimen is attributable to the genus *Delorhynchus*, we performed a phylogenetic analysis treating OMNH 77675 as a terminal taxon. We used the data matrix from MacDougall et al. [7] and were able to code various cranial characters for OMNH 77657. The resulting taxon-character matrix was subjected to a heuristic search under maximum parsimony, and branches with a minimum length of zero were set to collapse. Six optimal trees were obtained from the phylogenetic analysis, the strict consensus of which (Fig 2) recovers OMNH 77675 as the sister to *Delorhynchus cifellii*, supporting its assignment to the genus *Delorhynchus*. The *Delorhynchus* clade has a bootstrap support value of 65%, and a Bremer decay analysis shows that the clade collapses after the addition of one extra step. The clearly identifiable apomorphies of *Delorhynchus* that are present in OMNH 77675 include the distinctive anatomy of the dentition, and the anteroposteriorly expanded dorsal process of the maxilla. In addition, its assignment to *D*. *cifellii* rather than to *D*. *priscus* is supported by the absence of a distinct anterodorsal flange of the maxillary dorsal lamina, a feature that is present in *D*. *priscus*, but is absent in the new specimen and in the holotype and referred specimens of *D*. *cifellii* (Reisz et al. 2014).

## Discussion

The Richard Spur locality has produced many specimens of *Delorhynchus cifellii*, of which we are using seven, allowing us to ascertain relative ontogenetic ages using cranial features in combination with differences in size. The most distinctive feature that identifies OMNH 77675 as immature, and ontogenetically younger than the other known specimens, is the absence of prominent dermal tuberosities, which are found on the skull roof elements of larger individuals of *Delorhynchus cifellii* [4]. The lateral edges of the prefrontal, frontal, postfrontal, and the postorbital in OMNH 73362 are strongly ornamented with distinct dermal tuberosities, in addition to the circular pits and visible small dimples. This new specimen only exhibits the circular pitting and dimpling on its elements, and we interpret this as an indication of its young age. As previously discussed, the pattern of circular pits and dimples is not unique to *Delorhynchus*, being also found on several other parareptile taxa such as *Feeserpeton oklahomensis* [1]*, Colobomycter vaughni [5], Colobomycter pholeter* [11,13], *Microleter mckinzieorum* [3], *Macroleter poezicus* [14], and *Acleistorhinus pteroticus* [8]. What makes *Delorhynchus* particularly interesting is that there is an association between the size of the specimen, and the presence of prominent dermal ornamentation. The larger specimens have more pronounced tuberosities, whereas the smaller individuals have minor tuberosities or lack them entirely. It is because of the consistency of the relationship between size and robustness of ornamentation that we believe that this correlation is unlikely to be caused by sexual dimorphism, but rather, it is an ontogenetic characteristic developed over time [15]. This ontogenetic pattern is not unique to *Delorhynchus*, as shown by the cranial ontogeny of *Deltavjatia rossicus* and *Elginia mirabilis* [16], Late Permian pareiasaurian parareptiles. Juveniles of these taxa have less complex ornamentation than the adults, and the level of ornamentation and the rugosity of the sculpturing is correlated with size [16]. This phenomenon has been thoroughly studied in the extant members of crocodylomorpha where the ornamentation develops through reabsorption [15,17].

A classic character that is used to determine maturity of a specimen is the incomplete ossification and interdigitation of cranial sutures [18]. Sutures have long been used to determine maturity in amniotes, and this is because sutures function as growth sites for rapid bone expansion, and only remain functional in this role when not completely ossified [19–21]. The most rapid bone expansion is known to happen early in ontogeny [18,21]. This applies to most skeletal elements, and it is for this reason that sutures that are strongly interdigitated are linked to an organism that has completed the vast majority of its growth [18,21,22]. Thus, the lack of complete sutural contacts in OMNH 77675 is clearly indicative of immaturity. This is particularly apparent along the fronto-nasal suture, where the bones do not touch, and overlapping sutural surfaces are completely absent. In addition, the incomplete ossification of the sutures between many of the cranial elements facilitated disarticulation without damaging the individual elements. Interdigitation of sutures is independent of size, but correlated with ontogenetic age. The comparisons between the condition seen in the sub-adult *Feeserpeton* and the juvenile *Delorhynchus* specimen of similar size highlight this phenomenon, with the holotype and only known specimen of *Feeserpeton* having fully ossified sutures [1].

The relatively large temporal fenestra found in this specimen is another strong indicator of the juvenile state of this individual (Fig 1). A somewhat similar condition is seen in the Middle Permian milleretid parareptile, *Milleretta* from South Africa, which exhibits a temporal fenestra in a juvenile specimen and an unfenestrated condition in the larger specimen [23]. A temporal fenestra alone is not an indicator of early ontogenetic age, as many parareptiles exhibit temporal fenestration [2], including *Acleistorhinus pteroticus* [8], *Microleter mckinzieorum* [3], *Feeserpeton oklahomensis* [2], and *Colobomycter* [5,11]. Temporal fenestration is highly variable within Parareptilia, the diversity of temporal morphology includes variations of elements involved in fenestration, emargination, and even complete loss of fenestra [2,14,23]. This is in strong contrast to the condition seen in other amniote clades, in which the anatomy of the temporal fenestra tends to be conserved in closely related taxa, and evolutionary modifications of these openings are related to major events in the history of each clade [10].

There is very limited data on the ontogenetic variation of the temporal region in Palaeozoic diapsids and synapsids; the new evidence provided by the parareptile *Delorhynchus cifellii* indicates that the extensive variability in the size and shape of the temporal fenestra in this taxon is the result of ontogeny. Several specimens of *Delorhynchus cifellii* representing different ontogenetic stages have been found to decrease the size of the temporal fenestrae throughout ontogeny. This appears to occur through a combination of a posteriorly extended process of the jugal, a change in shape of the squamosal, and changes in the size of the quadratojugal and postorbital (Fig 3). This can be best seen in the temporal region of OMNH 73362 [4], where the posterodorsally directed process on the posterior edge of the jugal becomes so well developed that the upper portion of the temporal fenestra is reduced to a quarter of the size seen in the new specimen (Fig 3). The decrease in diameter of the temporal fenestra is correlated with the size of the specimen, making ontogeny the most likely cause for this change. The known morphological variation in skull anatomy due to growth, especially in the temporal region, allows us to place particular specimens more accurately within phylogenies [24] when its ontogenetic stage is taken into account.

The lateral temporal fenestra in *Delorhynchus cifellii* is not only reduced in size, but also likely divided into two (Fig 4), with the dorsal portion of the fenestra being larger in diameter (Fig 3). A division in the lateral temporal fenestra is also shown in the reconstruction of *Candelaria barbouri*. However, the bifurcation in *Candelaria* is achieved through a contact between the jugal, squamosal, and the quadratojugal, resulting in a fenestra and an emargination [25]. The dorsal portion of the lower temporal fenestra is reminiscent of the lateral temporal fenestra seen in *Milleretta rubidgei* [23], which is bordered by the same elements. The ventral portion of the temporal fenestra is bordered by only the jugal and quadratojugal with a small contribution of the squamosal. This lower portion of the fenestra would be similar in location, but not in shape or size to that found in *Belebey vegrandis* [26] and similar in relative location as that found in *Macroleter poezicus* [14]. However, in *Macroleter* and *Belebey*, the fenestra is clearly bordered by the jugal, quadratojugal, and squamosal.

In *Delorhynchus cifellii* the division of the original temporal fenestra is caused by the development of the posteroventral and posterodorsal processes of the jugal, which then presumably abuts against the squamosal and quadratojugal (Fig 4). Although the jugal exhibits the most drastic morphological change, the postorbital ventral process expands considerably to accommodate the growth of the jugal and maintains contact with the dorsal process of that bone. This means that the dorsal portion of the lateral temporal fenestra is now bordered by the squamosal, jugal, and postorbital.

It is interesting to note that the posteroventral and dorsal processes of the jugal are constants in all *Delorhynchus cifellii* specimens and likely developed first since they are found in the smallest specimen, and appear to change little in shape throughout ontogeny. The posteroventral process is of similar size and shape to that found in *Acleistorhinus pteroticus* [8] and plays a similar role in contacting the quadratojugal and contributing to the ventral border of the temporal fenestra. Although the posteroventral process continues to grow proportionately throughout ontogeny, the posterodorsal process appears to change gradually from a small protuberance that is found in the juvenile specimen OMNH 77675 (Fig 3A), to a robust emargination found on both OMNH 73362 and OMNH 73515 (Fig 3D and 3E).

Overall, this results in the subdivision and reduction of the lateral temporal fenestra (Fig 4). It is difficult to ascertain if the temporal fenestra would continue to reduce in size and eventually close, or close one portion of the divided temporal fenestra, or possibly even maintain a divided lower temporal fenestra. It is important to note that this subdivision in the lateral temporal fenestra of *Delorhynchus cifellii*, does not indicate a relationship with the condition seen in araeosceloids and other early diapsids, but is rather a testament to the variability found in the lower temporal fenestra among parareptiles [2]. Additionally, it is difficult to ascribe a functional advantage or explanation to the change seen in fenestration, with the anatomy of the dentition remaining comparatively constant throughout ontogeny. Although no extensive growth series is available for other Paleozoic fenestrated amniotes, the unfenestrated eureptile *Captorhinus aguti* is represented by numerous specimens that range in size from about 3–9 cm in skull length. Interestingly, the available evidence suggests that there is little change in sutural patterns of the skull roof of *Captorhinus aguti*, including the temporal region, but a thorough study of its ontogeny remains to be done. This is in strong contrast to the ontogenetic changes seen in *Delorhynchus cifellii*, where sutural patterns are variable not only in the temporal region but also in the snout, where the relationship between the maxilla and the lacrimal becomes more complex in the adult.

The contrast between the function of fenestration and change in dentition of two closely related taxa of Palaeozoic araeosceloids, *Petrolacosaurus* [27] and *Araeoscelis* [27], can give insight to functional purposes of secondary closure. *Petrolacosaurus* is characterized by the combination of a lightly built diapsid skull, and delicate, recurved marginal dentition. In contrast, its close relative *Araeoscelis* is characterized by the presence of a relatively more massive skull than in *Petrolacosaurus*, and a secondarily closed lower temporal fenestra, as well as comparatively massive, bulbous cheek teeth. Thus, the specialized crushing dentition of *Araeoscelis* could be related to the sturdy skull construction [27] and the closure of the temporal fenestra in this early diapsid reptile. The pattern of ontogenetic change in the temporal region of the reptile *Delorhynchus cifellii* provides a possible evolutionary mechanism for the closure of the temporal fenestra in reptiles like *Araeoscelis*, and other diapsids.

## Conclusions

Study of a new juvenile specimen of *Delorhynchus cifellii*, together with other specimens of this taxon, assists in better understanding parareptile ontogeny and evolution. This parareptile also contributes significantly to our knowledge of ontogenetic change in temporal fenestration among Palaeozoic amniotes, offering a more complete idea of possible cranial complexities and variations within a single taxon. In this particular case, we discovered ontogenetic shifts in the patterns of temporal fenestration from a single large fenestra in juveniles to a subdivided fenestra condition in larger individuals, which is caused by extensions of the surrounding elements of the temporal region. It is important to recognize that the known growth series of *Delorhynchus cifellii* is represented by only seven skulls, and is therefore far from complete. In addition, it is unclear if the largest specimen is a fully mature individual. It is because of this uncertainty that we acknowledge the possibility that full closure of the temporal fenestra may have occurred at some, currently unrecorded stage of its ontogeny. As previously shown, there is extensive evolutionarily independent variation in temporal patterns of fenestration in parareptiles [2].Within that context, it is important to understand the implications of this growth series of *Delorhynchus cifellii*, as it provides an additional level of complexity to temporal fenestration. Extensive variation in temporal fenestration is present not only between taxa of parareptiles, but also throughout the ontogeny of a single species. Ultimately, we see here a mechanism by which parareptiles and other amniotes can progressively reduce and possibly close their temporal fenestrae over the course of their ontogeny.

## Supporting Information

S1 Fig Cranial material of *Delorhynchus cifellii*. **(A)** dorsal and lateral views of the skull and lower jaw of OMNH 77675, **(B)** lateral view of anterior portion of the skull and lower jaw of OMNH 77676, **(C)** anterior portion of the skull and portion of the lower jaw of OMNH 77677, **(D)** Orbital region and posterior portion of the skull in dorsal and lateral view of OMNH 74722, **(E)** dorsal and lateral view of portions of the skull roof and jaw of OMNH 77678 **(F)** Lateral and dorsal views of the skull of the holotype of *Delorhynchus cifellii* OMNH 73515 **(G)** lateral and dorsal view of the a skull roof of OMNH 73362.
(DOCX)
