# Supplementary figures and images for: Ontogenetic Change in the Temporal Region of the Early Permian Parareptile Delorhynchus cifellii and the Implications for Closure of the Temporal Fenestra in Amniotes
Source: PLoS One. 2016 Dec 1;11(12):e0166819. doi: 10.1371/journal.pone.0166819 (PMC5132164; doi:10.1371/journal.pone.0166819)

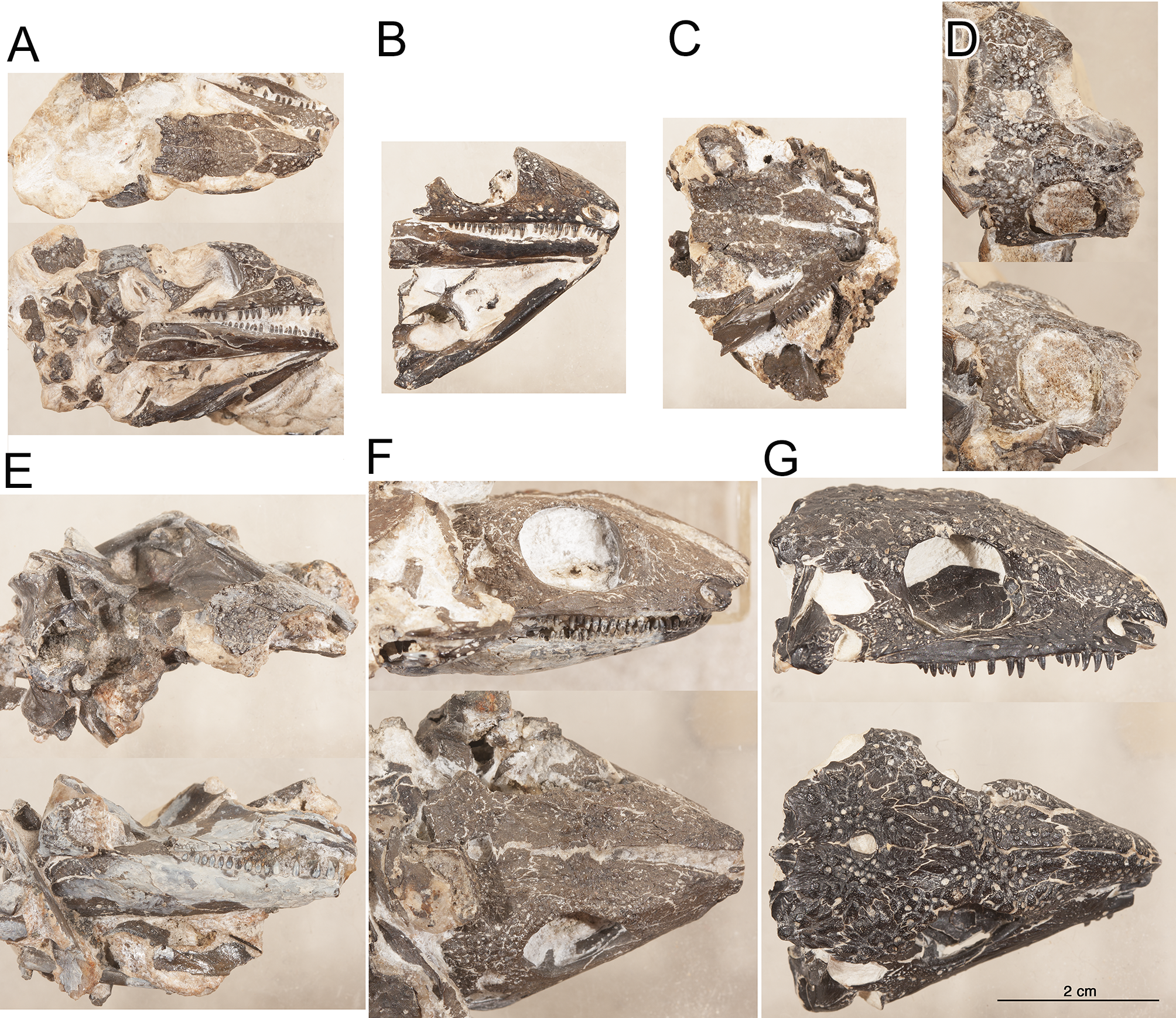

Supplement: S1 Fig — (A) dorsal and lateral views of the skull and lower jaw of OMNH 77675, (B) lateral view of anterior portion of the skull and lower jaw of OMNH 77676, (C) anterior portion of the skull and portion of the lower jaw of OMNH 77677, (D) Orbital region and posterior portion of the skull in dorsal and lateral view of OMNH 74722, (E) dorsal and lateral view of portions of the skull roof and jaw of OMNH 77678 (F) Lateral and dorsal views of the skull of the holotype of Delorhynchus cifellii OMNH 73515 (G) lateral and dorsal view of the a skull roof of OMNH 73362. (DOCX) [file pone.0166819.s001.docx]
